# Global trends and hotspots of inflammation in diabetic retinopathy: a literature review and bibliometric analysis

**DOI:** 10.3389/fmed.2025.1615045

**Published:** 2025-09-18

**Authors:** Jijuan Zhong, Chensi Yao, Yamei Jin

**Affiliations:** Department of Endocrinology, The First Affiliated Hospital of Zhejiang Chinese Medical University (Zhejiang Provincial Hospital of Chinese Medicine), Hangzhou, China

**Keywords:** diabetic retinopathy, inflammation, VOSviewer, CiteSpace, bibliometric analysis

## Abstract

**Background:**

Diabetic retinopathy (DR) is a global public health problem, with inflammation playing a pivotal role in its progression. In this study, we aimed to assess the current research landscape of inflammation in DR and identified emerging frontiers using bibliometric analysis.

**Methods:**

Relevant publications were retrieved from the Web of Science Core Collection database, and VOSviewer and CiteSpace were used for bibliometric analysis and visualization.

**Results:**

Overall, 3,419 publications on inflammation in DR over the past 44 years were identified, exhibiting an upward trend. China had the highest number of publications, while the United States had the majority of citations. Shanghai Jiao Tong University was the most active institution, and *Investigative Ophthalmology Visual Science* was the most productive journal. Timothy S. Kern contributed the most publications, with the highest total/average citations. Research mainly focused on the risk factors, mechanisms, and potential therapies in this field. Key areas of future exploration include the roles of the NLRP3 inflammasome and gut microbiota, the correlation between DR and age-related macular degeneration, and advancements in identification techniques and optical coherence tomography.

**Conclusion:**

We provide a systematic overview of the academic literature on inflammation in DR over the past few decades. The United States and China have been pivotal in conducting research in this field. Optical coherence tomography screening and the precise identification of inflammation in DR are likely to emerge as the next area of focus. Further understanding the roles of NLRP3 and the gut microbiota in inflammation in DR is also a potential research direction. Additionally, identifying the mechanisms of inflammation underlying DR and age-related macular degeneration is a cutting-edge and urgent research priority.

## 1 Introduction

Diabetic retinopathy (DR), as a vision-threatening microvascular complication of diabetes mellitus (DM), is the leading cause of vision loss among the working-age population (1, 2). With the rising prevalence of DM, DR is growing by leaps and bounds, becoming a public health issue. The prevalence of DR in 2020 was estimated to be 103.12 million, which is expected to rise to 130 million in 2030 and 161 million in 2045 (3).

DR is characterized as a microvascular complication of DM as well as a neurodegenerative disease (4). Retinal microvascular endothelial cells (RMECs), together with pericytes, neurons, glial cells, and professional immune cells, constitute the retinal neurovascular unit (NVU) (4). Chronic hyperglycemia in a diabetic milieu may impair the integrity of the NVU, leading to damage of the blood–retina barrier (BRB), neovascularization, neuroinflammation, neuronal death, and gliosis (4). It is widely acknowledged that DM often exhibits low-grade inflammation. Furthermore, there is growing evidence showing that low-grade inflammation is also significantly associated with DR (5). Various types of retinal cells, including RMECs, microglia, Müller glia, astrocytes, etc., are activated in DM; they secrete inflammatory mediators, participating in the chronic inflammation that triggers microvascular injury and neurodegeneration in DR (5). For example, long-term hyperglycemia can alter the ramified microglial cells into an amoeboid shape, changing them from an anti-inflammatory (M2) to a pro-inflammatory (M1) phenotype, which results in the release of various pro-inflammatory cytokines and subsequent damage to the NVU and BRB (6). Furthermore, chronic hyperglycemia can disrupt the antioxidant defenses of the retina, contributing to the production of large amounts of reactive oxygen species (ROS), which then trigger the classic pathological mechanisms of DR, including the polyol pathway, advanced glycation end products (AGEs) accumulation, the protein kinase C pathway, and the hexosamine pathway (7). Oxidative stress, one of the reasons for “metabolic memory” in the Diabetes Control and Complications Trial (DCCT) (8), can provoke a large number of inflammatory mediators, such as tumor necrosis factor-α (TNF-α), intercellular adhesion molecule-1 (ICAM-1), interleukin-6 (IL-6), interleukin-8 (IL-8), monocyte chemotactic protein-1 (MCP-1), and cyclooxygenase 2 (COX-2) via the nuclear factor-kappa-B (NF-𝜅B) pathway. In addition, oxidative stress can upregulate various angiogenic factors, such as vascular endothelial growth factor (VEGF), stromal cell-derived factor-1, angiopoietin, and erythropoietin, via the HIF-1 pathway, resulting in retinal vascular inflammation and neurodegeneration (7, 9). Meanwhile, the metabolic abnormalities caused by high glucose can activate the NOD-like receptor protein 3 (NLRP3) inflammasome, which stimulates self-cleavage and triggers procaspase-1, inducing the release of the pro-inflammatory cytokines IL-1β/18 to further promote the inflammatory response (10).

Clinically, the levels of multiple pro-inflammatory mediators, such as TNF-α, IL-1β, MCP-1, and ICAM-1, are higher in the vitreous humor, aqueous humor, and retina of patients with DR (11, 12). These cytokines, chemokines, and adhesion molecules interact with each other to construct a complicated molecular network that further exacerbates the inflammatory environment of DR (13). In addition, clinical and experimental evidence have demonstrated that targeting inflammation may prevent or delay disease progression and ameliorate visual acuity in patients with DR, indicating that inflammation is a promising therapeutic target in DR (5).

Intravitreal anti-VEGF therapy is the first-line treatment for DR, with certain agents approved by the United States Food and Drug Administration (FDA) for specific stages, while others may be used off-label. Laser photocoagulation remains an important option, used both as a primary therapy and as an adjunct in the management of DR (14). Researchers have demonstrated that anti-VEGF therapy provides a significant benefit for patients with center-involved diabetic macular edema (CI-DME), which proved more effective than laser photocoagulation in improving visual acuity (15, 16). However, the American Academy of Ophthalmology Preferred Practice Pattern guidelines recommend focal or grid laser photocoagulation as the preferred treatment for non-center-involved DME (NCI-DME) (14). In addition, corticosteroids remain an important therapeutic option for DME, as they not only antagonize VEGF activity but also suppress inflammatory cytokines, reflecting their potent anti-inflammatory properties (17, 18). However, they all have some notable limitations. For example, anti-VEGF therapy often fails to restore vision fully, and frequent intravitreal injections may result in endophthalmitis (19, 20). Laser photocoagulation can cause retinal scars, choroidal detachment, tardy dark adaptation, peripheral visual field damage, and even macular edema (21). Finally, intravitreous steroid therapy is associated with an increased risk of intraocular pressure and cataract formation (17, 22). Therefore, a deeper understanding of the fundamental mechanisms of DR is urgently needed to develop more effective and safer therapeutic strategies.

Before advancing further basic and clinical research, it is imperative to comprehensively understand the current research trends and frontiers on inflammation in DR. Although the role of inflammation in DR has attracted increasing attention from researchers and many articles have been published, an overall review of this topic is lacking. Recently, bibliometric studies and visualization analysis have been identified as crucial in helping scholars gain a global perspective on a specific scientific research area, including medicine (23). Bibliometric analysis is a statistical method that reveals both qualitative and quantitative information about countries, regions, institutions, journals, authors, keywords, the impact of the work, and the collaborative network within the complex body of existing publications, offering a comprehensive overview of the field (24).

We conducted a bibliometric study to map out a systematic overview of the academic literature on inflammation in DR between 1 January 1981, and 21 May 2024. This study aimed to explore the current state of development, research hotspots, and future trends of DR-related inflammation, addressing the existing gaps in this field. To the best of our knowledge, this is the first bibliometric analysis on this topic.

## 2 Materials and methods

### 2.1 Data source and search strategy

The Science Citation Index Expanded of the Web of Science Core Collection (WOSCC) database, widely regarded as the most authoritative and credible academic source for bibliometric analysis (25), was utilized in this study. All data were searched on 21 May 2024. According to previous studies (26–29), the search terms are as follows:

1. TS = (“inflammation” OR “inflammations” OR “inflammatory”).
2. TS = (“diabetic retinopathy”).
3. #1 AND #2.

**Figure 1:**
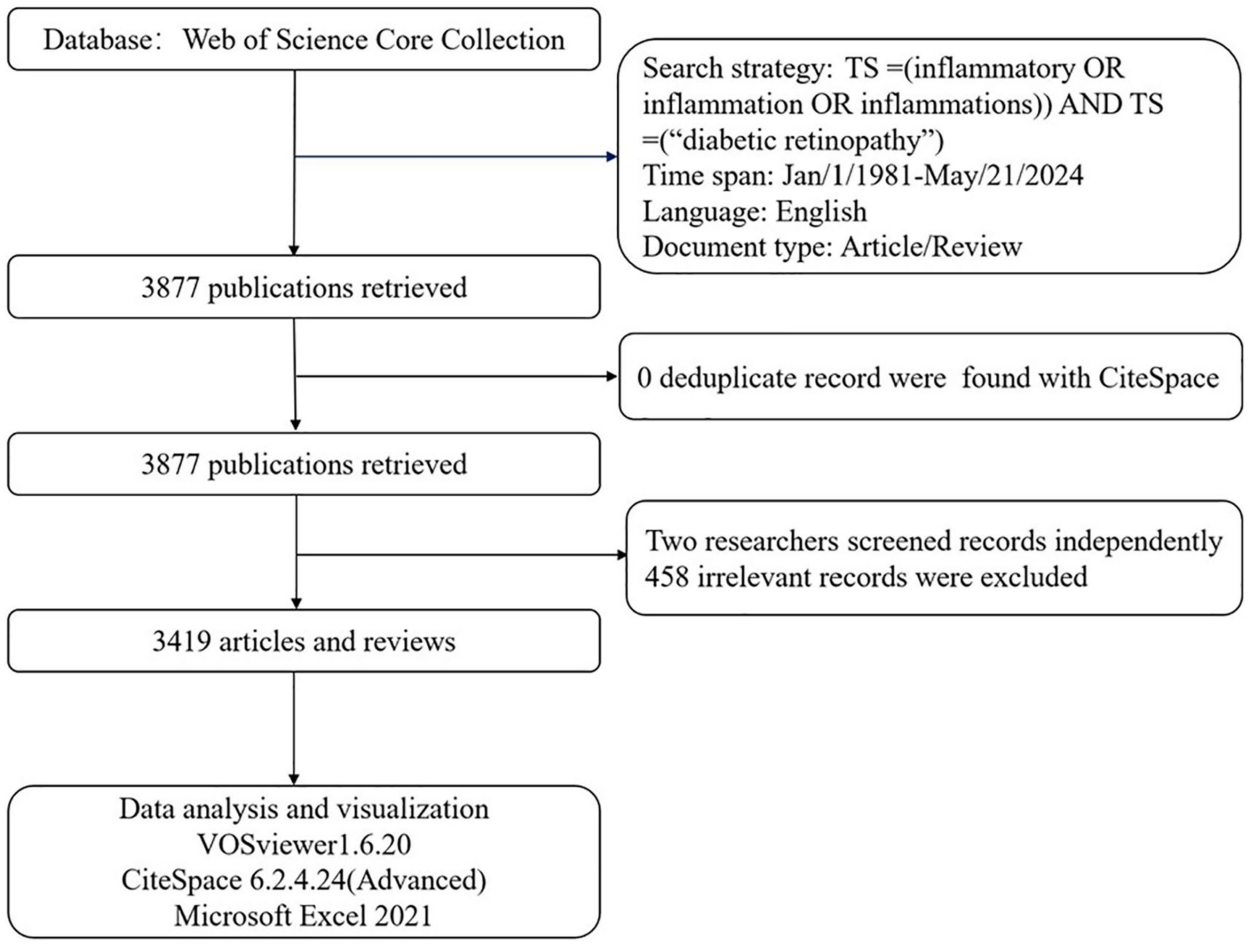
Flowchart of data collection and study design.

The time span of the publications was from 1 January 1981, to 21 May 2024. The literature consisted of journal articles and reviews, with language limited to English. In total, 3,877 publications met the criteria after duplicates were removed using CiteSpace. These publications were independently reviewed by two researchers (JZ and YJ), and differences in opinion were resolved by a third researcher (CY). Finally, 3,419 publications, comprising 2,583 articles and 836 reviews, were included in this bibliometric analysis; 458 irrelevant publications were excluded following manual screening. Eligible publications, including full records and cited references, were exported and saved as plain.txt files for the bibliometric analysis (Figure 1). However, to maintain conciseness, we only cited recent key advances (2020–2024) as well as classic articles to acknowledge the foundational work. The research was in accordance with the ethics committee of The First Affiliated Hospital of Zhejiang Chinese Medical University (Zhejiang Provincial Hospital of Chinese Medicine) with the 1964 Helsinki Declaration.

### 2.2 Data analysis and visualization

Microsoft Excel (Version 2021), VOSviewer (Version 1.6.20), and CiteSpace (Version 6.2.4.24, advanced) were used for data analysis and visualization.

Microsoft Excel was used to organize data, including the number of publications and citations, for the study’s statistical charts.

VOSviewer, first developed by van Eck and Waltman at Leiden University in 2009, is now applied in a wide range of fields to depict bibliometric maps through collaborative network analysis, co-occurrence analysis, and cluster analysis (30). In this study, we utilized VOSviewer to visualize collaboration networks among high-ranked countries, institutions, authors, journals, and keywords.

CiteSpace, developed by Professor Chaomei Chen of Drexel University, is a widely used software package for bibliometric analysis, particularly for investigating the research status, hotspots, and forecast frontiers over time (31). We used CiteSpace to assess keyword clusters and burst keywords from published studies.

A thesaurus text file was created to avoid synonyms, such as “type 2 diabetes” and “type 2 diabetes mellitus” in the keyword diagram. The threshold for principal countries/regions, institutions, journals, authors, and keywords in this research was established based on actual data to gain a precise and intuitive understanding of the field of DR with inflammation.

## 3 Results

### 3.1 General statistics

Overall, a total of 3,419 publications met the search criteria, comprising 2,583 (75.55%) articles and 836 (24.45%) reviews. These studies were performed by 14,501 authors from 3,252 institutions in 80 countries. They were published in 804 journals and cited 147,489 times, with an average citation of 43.14 per paper.

**Figure 2:**
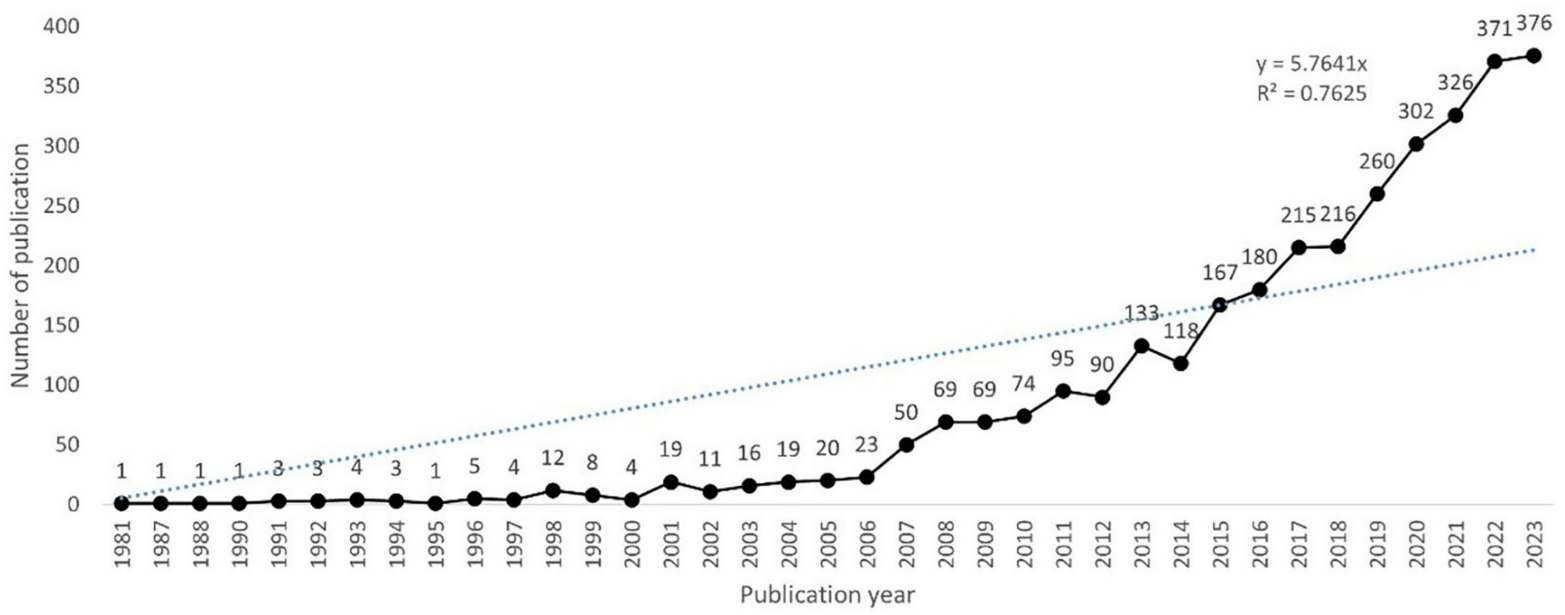
Trends in the annual number of publications.

As shown in Figure 2, the first study on inflammation in DR was published in 1981, and there were fewer than 20 papers per year before 2005. However, since 2006, this number has increased steadily, exceeding 200 in 2017 and reaching a peak of 376 papers in 2023. A polynomial fitting curve was constructed, showing a rising trend that was highly correlated with the year of publication (*R*^2^ = 0.7625). This result suggests that the role of inflammation in DR has attracted increasing interest from scholars, making it a research hotspot.

### 3.2 Distribution of countries/regions

There were 80 countries that published papers on inflammation in DR. Table 1 presents the top 10 productive countries, with China having the highest number of publications (1,127, 32.96%), followed by the US (994, 29.07%) and Italy (191, 5.59%). The US had the majority of citations (48,528), followed by China (23,826) and Germany (8,411). Germany ranked first (58.41) in terms of average citations per publication, followed by the US (48.82) and the United Kingdom (48.71).

**Table 1:** The top 10 most productive countries.

Rank	Country	C	*P* (%)	CF	ACI	Total link strength
1	China	1,127	32.96	23,826	21.14	248
2	United States	994	29.07	48,528	48.82	556
3	Italy	191	5.59	6,121	32.05	139
4	Japan	189	5.53	7,839	41.48	87
5	India	175	5.12	3,569	20.40	94
6	United Kingdom	158	4.62	7,696	48.71	207
7	Germany	144	4.21	8,411	58.41	151
8	Spain	106	3.10	4,593	43.33	87
9	Australia	100	2.92	4,690	46.90	152
10	South Korea	87	2.54	2,048	23.54	52

C, count; P, proportion; CF, citation frequency; ACI, average citation per item.

**Figure 3:**
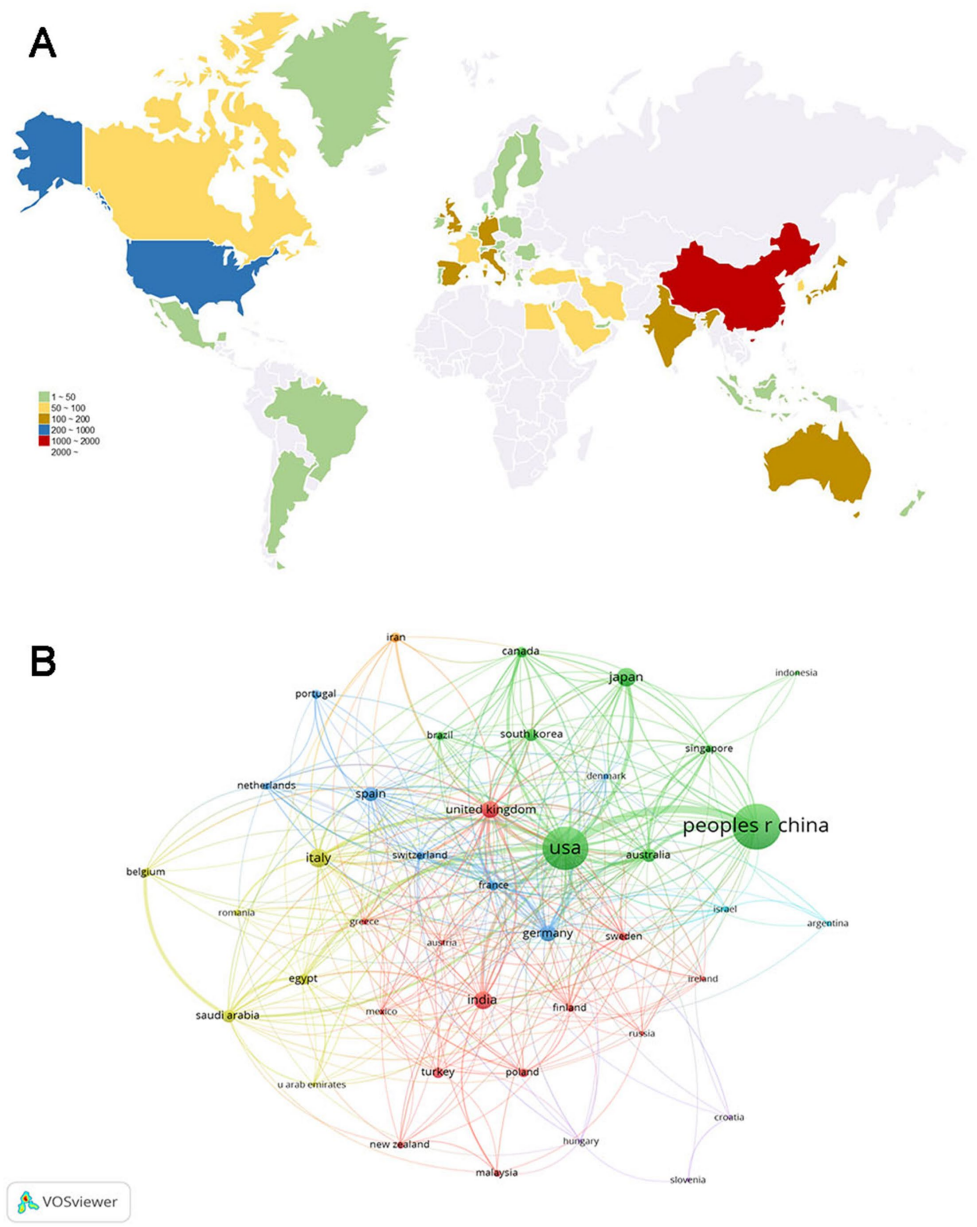
Analysis of country/region. **(A)** Geographical distribution of global publications. The volume of publications is represented by color variation. **(B)** Collaboration analysis of countries/regions. The nodes of different colors represent countries/regions with distinct clusters, and the thickness of the lines indicates the intensity of cooperation.

We also assessed the distribution and cooperation of countries to evaluate the international cooperation network structure. As shown in Figure 3A, the volume of publications is represented by color variation; the countries involved in this field were primarily distributed in East Asia, North America, Europe, and Oceania. We established a minimum requirement of 10 publications per country; a total of 82 countries were selected to construct the collaboration diagram (Figure 3B). Each node represents a country, with its size indicating the number of publications. Each link represents a collaborative relationship, with the thickness indicating the depth of collaboration. The US had the strongest total link strength (556) for co-authorship links with other countries, whereas China (248) and the United Kingdom (207) ranked second and third, respectively, suggesting that these three countries worked closely with other countries/regions in the research domain.

### 3.3 Analysis of institutions and authors

A total of 14,501 authors from 3,252 affiliations contributed to the research on inflammation in DR. Table 2 lists the affiliations with the most publications in this field. Except for King Saud University, all were from the 10 most productive countries. Shanghai Jiao Tong University had the most publications (83, 2.43%), which was followed by Sun Yat-Sen University (80, 2.34%) and Case Western Reserve University (77, 2.25%). Case Western Reserve University (6,185) had the most citations, whereas Harvard University (115.49) ranked the top based on the average citations per publication.

**Table 2:** The top 10 most productive institutions.

Rank	Institution	Country	C	*P* (%)	CF	ACI	Total link strength
1	Shanghai Jiao Tong Univ	China	83	2.43	1,809	21.80	40
2	Sun Yat Sen Univ	China	80	2.34	2,586	32.33	40
3	Case Western Reserve Univ	United States	77	2.25	6,185	80.32	41
4	Univ Oklahoma	United States	61	1.78	3,659	59.99	41
5	Wayne State Univ	United States	54	1.58	2,763	51.17	10
6	Nanjing Med Univ	China	50	1.46	2,354	47.08	26
7	Univ Catania	Italy	48	1.40	1,485	30.94	0
8	Harvard Univ	United States	47	1.37	5,428	115.49	15
9	Univ Michigan	United States	43	1.26	2,023	47.05	22
9	Queens Univ Belfast	United Kingdom	43	1.26	2,015	46.86	10
9	King Saud Univ	Saudi Arabia	43	1.26	1,002	23.30	21
9	Tianjin Med Univ	China	43	1.26	784	18.23	8
10	Univ Wisconsin	United States	41	1.20	1,606	39.17	32

As shown in Table 3, Timothy S. Kern from the University of California, Irvine, contributed the most publications (50, 1.46%), followed by Jian-xing Ma from Wake Forest University (39, 1.14%) and Maria B. Grant from the University of Alabama, Birmingham (32, 0.94%). Furthermore, Timothy S. Kern had the highest number of citations and average citations, demonstrating his significant academic influence in this field.

**Table 3:** The top 10 most productive authors.

Rank	Author	Institution	C	*P* (%)	CF	ACI	Total link strength
1	Timothy S. Kern	Univ of California-lrvine	50	1.46	4,014	80.28	58
2	Jian-xing Ma	Wake Forest Univ	39	1.14	1,932	49.54	9
3	Maria B. Grant	Univ of Alabama at Birmingham	32	0.94	1,277	39.91	18
4	Claudio Bucolo	Univ of Catania	31	0.91	1,092	35.23	32
5	Ahmed M. Abu El-Asrar	King Saud University	30	0.88	612	20.40	87
6	Jena J. Steinle	Wayne State Univ	29	0.85	615	21.21	34
7	Rafael Simo	Univ Autònoma de Barcelona	24	0.70	1,425	59.38	27
7	Filippo Drago	Univ of Catania	24	0.70	957	39.88	32
8	Cristina Hernandez	Univ Autònoma de Barcelona	22	0.64	668	30.36	27
9	Mohamed AlShabrawey	Oakland Univ	21	0.61	825	39.29	10
9	Ghislain Opdenakker	King Saud Univ	21	0.61	416	19.81	71
10	Yunpeng Du	Univ of California-Irvine	20	0.58	1,245	62.25	43
10	Mohammad Mairaj Siddiquei	King Saud Univ	20	0.58	453	22.65	70

**Figure 4:**
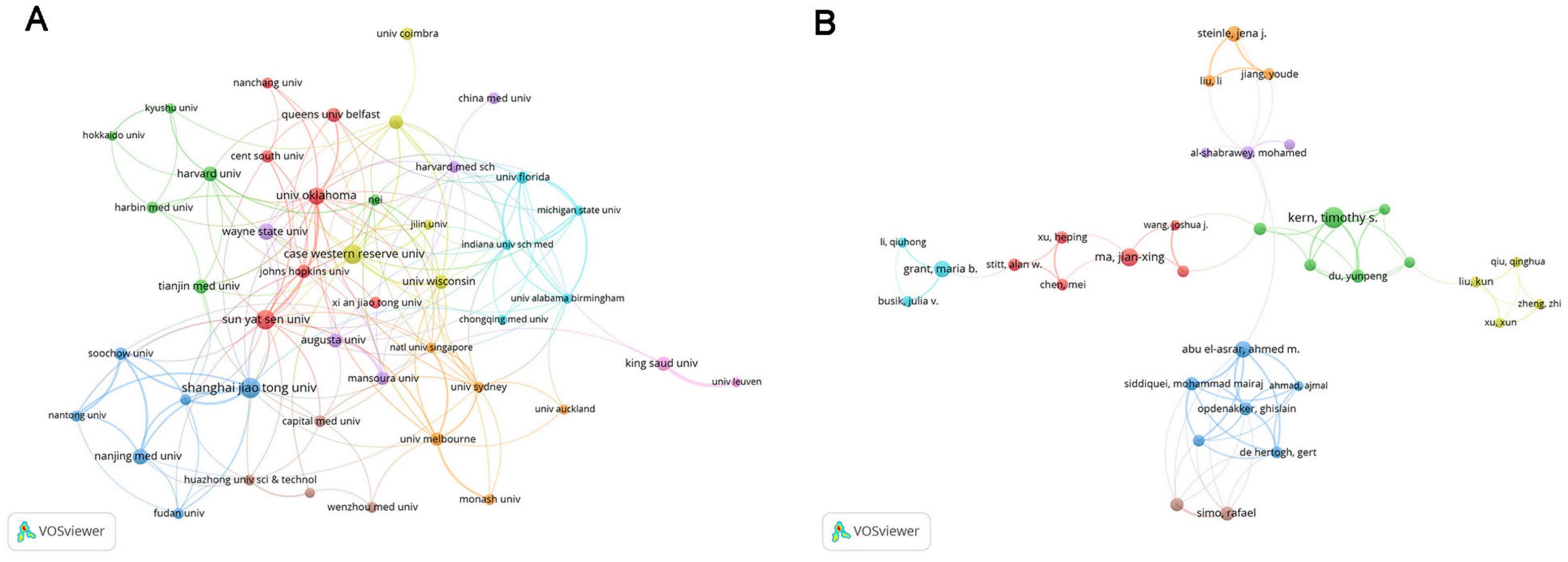
Analysis of institutions and authors. **(A)** Inter-institutional collaboration analysis. The nodes of different colors represent institutions within different clusters, and the thickness of the lines indicates the intensity of cooperation. **(B)** Collaboration analysis of authors. The nodes of different colors represent authors belonging to different clusters, and the thickness of the lines indicates the intensity of cooperation.

The co-occurrence network of institutions and authors was also explored by applying a minimum publication threshold of 20/12 documents for each institution/author, respectively (Figure 4). The size of the nodes represents the number of publications, and the thickness of the lines indicates the intensity of cooperation. However, the collaborative relationships among them seemed to be poor, which points to the need for closer collaboration in the future.

### 3.4 Analysis of journals

**Figure 5:**
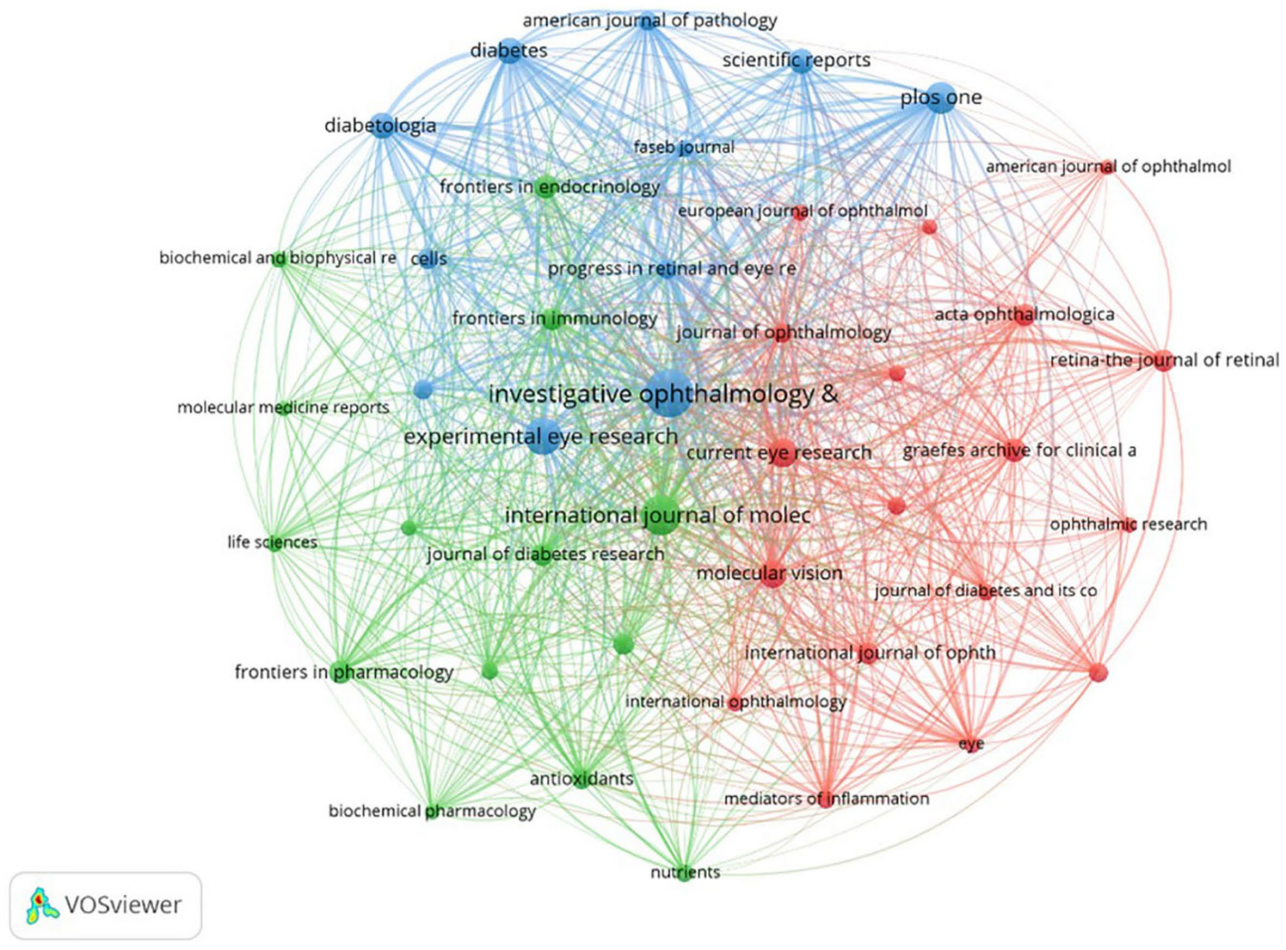
Collaboration analysis of journals. The nodes of different colors represent journals belonging to different clusters, and the thickness of the lines indicates the intensity of cooperation.

Studies on inflammation and DR research were published across 804 journals. By the criterion that each journal published a minimum of 16 papers, a total of 43 journals were selected to generate a collaborative network map (Figure 5). Each node represents a journal, with its size proportional to the number of publications it has produced. The thickness of the connecting lines indicates the strength of cooperation between the journals. Each color represents a specific research direction cluster, divided into three clusters.

Table 4 displays the top 10 most prolific journals, which account for approximately 21% (715/3419) of the total publications. Among them, five publishers were from the US, while two were from the United Kingdom and Germany. *Investigative Ophthalmology Visual Science* led with 155 publications, followed by the *International Journal of Molecular Sciences* with 110 publications and *Experimental Eye Research* with 94 publications. In terms of impact factor (IF; 2023), *Diabetologia* ranked first (8.4), followed by *Diabetes* (6.2), which had the highest average citations per paper (80.89), and *Investigative Ophthalmology Visual Science* (5.0).

**Table 4:** The top 10 most productive journals.

Rank	Journal	Country	IF 2023	C	*P* (%)	CF	ACI	Total link strength
1	Investigative Ophthalmology Visual Science	United States	5.0	155	4.53	7,069	45.61	1,469
2	International Journal of Molecular Sciences	Switzerland	4.9	110	3.22	2,936	26.69	1,113
3	Experimental Eye Research	United States	3.0	94	2.75	2,338	24.87	550
4	Plos One	United States	2.9	70	2.05	2,401	34.30	638
5	Current Eye Research	United Kingdom	1.7	57	1.67	1,266	21.51	427
6	Molecular Vision	United States	1.8	54	1.58	2,024	37.48	463
7	Diabetologia	Germany	8.4	48	1.40	2,332	48.58	472
8	Diabetes	United States	6.2	46	1.35	3,721	80.89	662
9	Scientific Reports	United Kingdom	3.8	42	1.23	1,068	25.43	291
10	Graefes Archive for Clinical and Experimental Ophthalmology	Germany	2.4	39	1.14	1,500	38.46	309

### 3.5 Analysis of keywords

**Figure 6:**
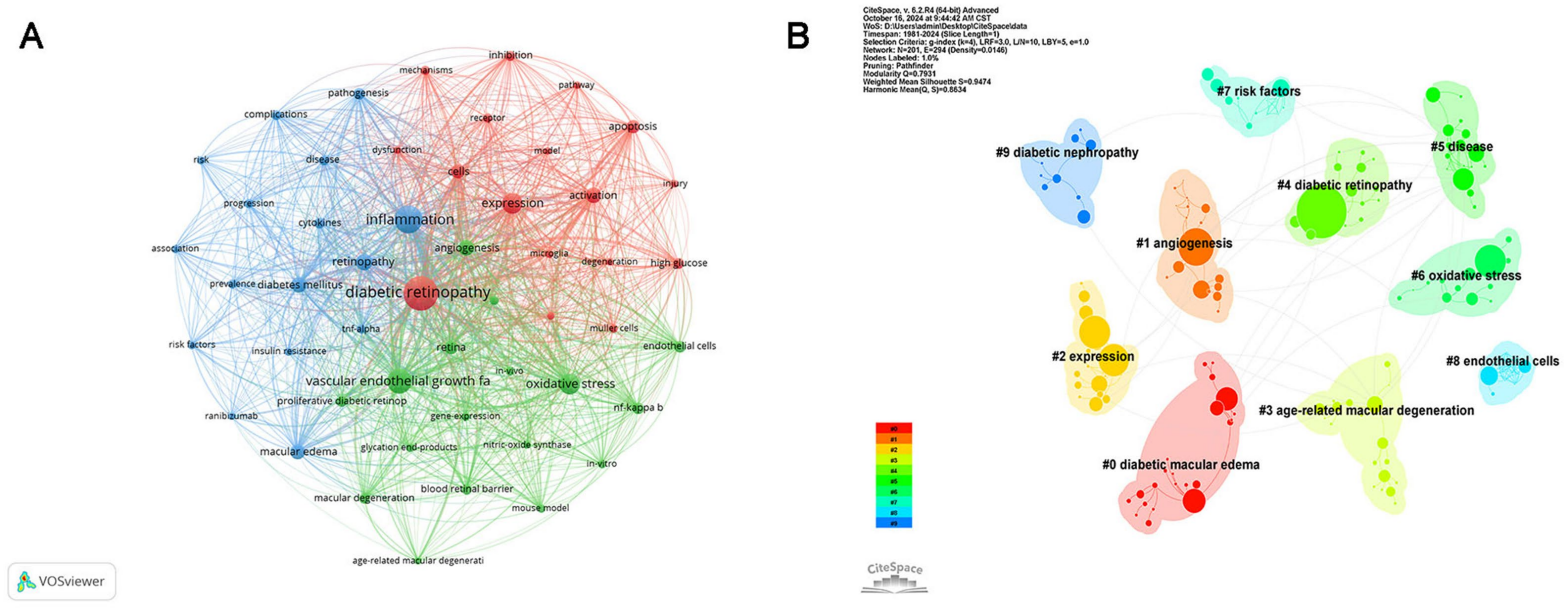
Co-occurrence analysis of keywords. **(A)** Network visualization of keyword co-occurrence. The nodes of different colors represent keywords organized into distinct clusters, and the thickness of the lines indicates the degree of cooperation between them. **(B)** Cluster analysis of keywords: divided into 10 clusters by different colors.

VOSviewer was used to analyze the keyword co-occurrence of all keywords from 3,419 publications. Figure 6A displays the keyword co-occurrence network, which includes 50 keywords with a minimum occurrence frequency of 90 times. Each node represents a keyword, with its size corresponding to the frequency of occurrence, while each line symbolizes the relationship between them. The keywords related to inflammation in DR were categorized into three clusters, with different colors representing distinct research interests. First, the red cluster mainly concentrated on the role of inflammation in cell damage, with high-frequency keywords including “diabetic retinopathy,” “injury,” “activation,” “inhibition,” “apoptosis,” “high glucose,” “mechanisms,” “microglia,” “Müller cells,” and “neurodegeneration.” The green cluster emphasized the mechanisms of neovascularization, with “vascular endothelial growth factor” ranked first, followed by “oxidative stress,” “angiogenesis,” “retina,” “retinal endothelial cells,” “nf-kappa b,” “proliferative diabetic retinopathy,” “macular degeneration,” and “blood retinal barrier” sequentially. The blue cluster included the prevalence, risk factors, and pathogenesis of DR. CiteSpace was also used to perform cluster analysis; 10 clusters were generated, namely diabetic macular edema, angiogenesis, expression, age-related macular degeneration (AMD), DR, disease, oxidative stress, risk factors, endothelial cells, and diabetic nephropathy (Figure 6B).

**Figure 7:**
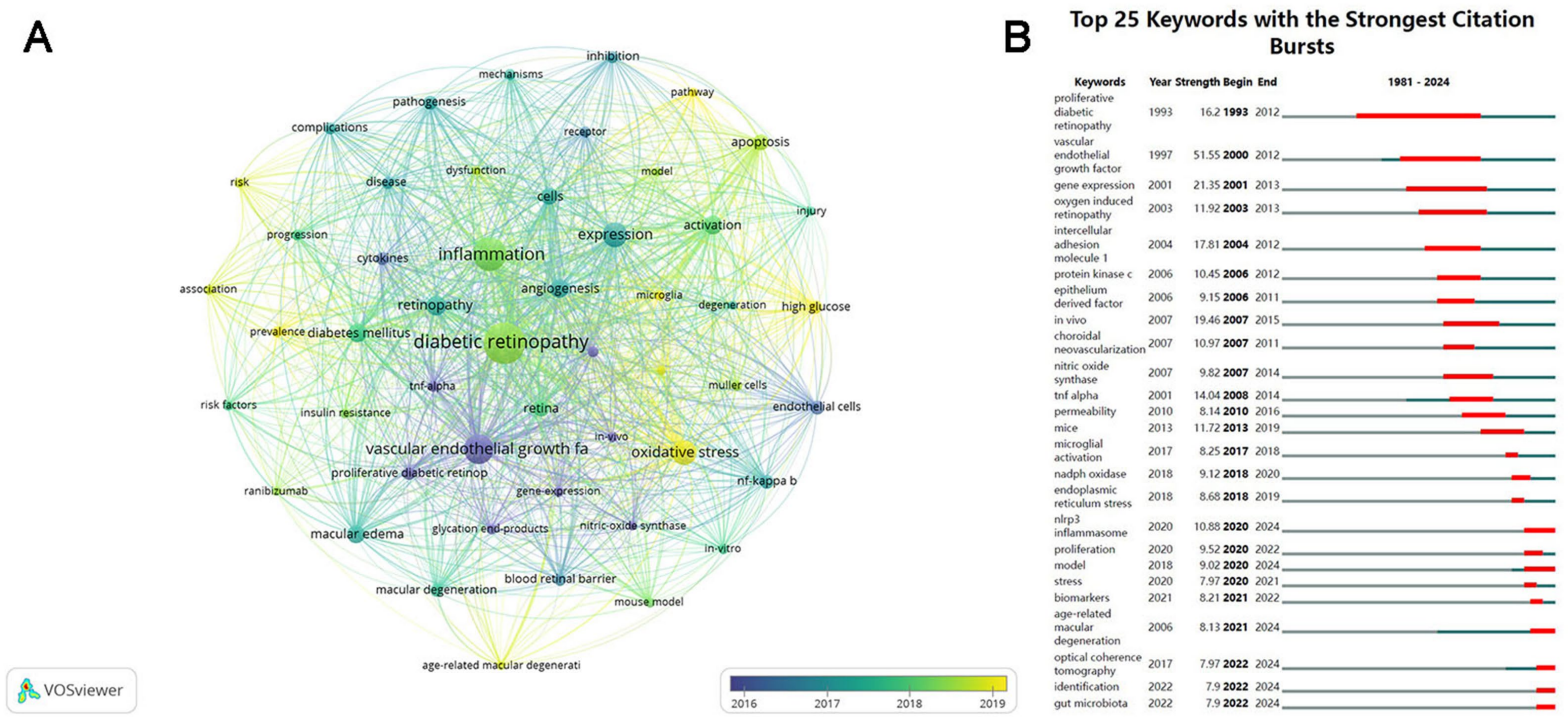
Visualization of the keywords over time. **(A)** Visualization of the keyword co-occurrence network according to the average years of publication (blue: earlier, yellow: later). **(B)** Top 25 keywords with the strongest citation bursts. The blue lines represent the base timeline, and the red lines indicate the period of the keyword bursts.

Figure 7A illustrates the evolution of keywords, where each node represents a keyword and is color-coded according to its average year of publication. The most frequent keywords emerged after 2016, including oxidative stress, microglia, pathway, prevalence, and AMD, which replaced VEGF, proliferative DR, endothelial cells, neovascularization, and gene expression, becoming hotspots in the research area of inflammation and DR. Figure 7B displays the top 25 keywords with the strongest citation bursts. “Vascular endothelial growth factor” had the maximum strength (51.55) and was a study hotspot from 2000 to 2012. “Proliferative DR” had the longest outbreak duration (1993–2012) with a strength of 16.77. Meanwhile, “NLRP3 inflammasome” and “model” from 2020, “age-related macular degeneration” from 2021, and “optical coherence tomography,” “identification,” and “gut microbiota” from 2022 have become the current research focuses in this field.

## 4 Discussion

### 4.1 General information

In this study, a bibliometric analysis was conducted to identify the structural associations and research hotspots on DR-related inflammation using the WOS database via VOSviewer and CiteSpace. In total, 3,419 English articles and reviews from 1 January 1981, to 21 May 2024, were compiled. The annual publication trend was generally upward, as indicated by the fitting curve (Figure 2). The field of inflammation in DR received little attention before 2005, as fewer than 20 publications occurred per year. Subsequently, a rapidly developing trend emerged, with the annual publication volume exceeding 200 articles after 2017 and reaching over 300 articles after 2020. The rising prevalence and incidence of DM has been partially attributed to the remarkable increase in DR diagnosis. Furthermore, recent advancements in diagnosis, therapeutic approaches, and public awareness of DR over the past few decades have contributed to progress in this academic field.

As shown in Figure 3, the countries participating in this research field were primarily located in East Asia, North America, Europe, and Oceania. China (1,127 papers) and the US (994 papers) were the top two high-yield countries, accounting for 62.04% of the total publications. Based on the total link strength, the US had the highest number of academic collaborations among countries. As expected, the majority of the top 10 productive institutions were from the US and China, indicating their dominant status in the field. Furthermore, the top 10 journals were published in the US and Europe. *Investigative Ophthalmology Visual Science* emerged as the journal with the highest number of publications, whereas *Diabetes* ranked first in terms of average citations, both of which are located in the US. Whether in consideration of collaborations among countries, institutions, or journals, there was a preference for the US, demonstrating its outstanding contributions to this academic field.

Among the top 10 most productive authors, Professor Timothy S. Kern from the University of California, Irvine, ranked first in both the number of publications (50) and the average number of citations per paper (80.28). His research has focused on elucidating the inflammatory processes in the early stages of DR, including interactions between leukocytes, photoreceptor cells, Müller cells, macrophages, microglia, and RMECs (32), the role of CD40 (33), and contributions of inflammatory mediators, such as ICMA-1, IL-17A, and inducible nitric oxide synthase (iNOS), to retinal capillary dysfunction or neurodegeneration (32, 34, 35). He has also searched potential therapeutic methods targeting inflammation, including inhibitors of AGEs and aldose reductase, salicylate-based anti-inflammatory drugs, and photobiomodulation (PMB), which may represent promising novel treatments for DR (32, 34, 36). Nevertheless, collaboration among international and domestic institutions and authors remains limited, underscoring the urgent need for greater academic cooperation to overcome knowledge barriers and address shared challenges. In this study, we employed keyword co-occurrence analysis (Figure 6) and keyword burst analysis (Figure 7) to identify research hotspots and frontiers in the field.

### 4.2 Research hotspots

Keyword co-occurrence analysis highlighted a strong association between DR and inflammation. Among the most commonly used keywords besides “DR” and “inflammation,” “vascular endothelial growth factor” ranked third. In addition to hypoxia chronic hyperglycemia AGEs and pro-inflammatory cytokines can stimulate VEGF gene expression (37). VEGF has been identified as a key factor in retinal neovascularization diabetic macular edema (DME) and even vitreous hemorrhage and tractional retinal detachments (38). In late-stage DR VEGF drives capillary proliferation leading to neovascularization a characteristic of proliferative DR (PDR). Notably VEGF upregulation has also been observed in the early stages of DR linking neurodegeneration to microvascular injury through BRB breakdown (39, 40). Currently anti-VEGF therapy represents the first-line treatment for vision-threatening DME and has also demonstrated efficacy in managing PDR (41). Corticosteroids remain an important therapeutic option for DME particularly in cases refractory to laser photocoagulation not only by suppressing VEGF expression but also by attenuating VEGF induction through multiple pro-inflammatory mediators (5, 42).

Another frequently occurring keyword was “oxidative stress.” Evidence from the DCCT demonstrated that intensive glycemic control in the early stages of diabetes conferred long-term protective effects on DR progression, persisting even a decade after the trial. This “metabolic memory” or “legacy effect” is considered to be mediated, at least in part, by oxidative stress (8). Hyperglycemia drives the excessive production of mitochondrial ROS, resulting in oxidative stress and mitochondrial dysfunction. Damaged mitochondria then serve as a continuous source of ROS, a process that persists even after normalization of glycemia (7). Increased oxidative stress, in turn, activates classical pathological mechanisms of DR, including the polyol pathway, AGEs accumulation, the protein kinase C pathway, and the hexosamine pathway (7). AGEs accumulation can also upregulate the expression of cytokines and adhesion molecules via the NF-𝜅B pathway and interact with the innate immune system by activating toll-like receptor 4 (TLR-4) (43). Furthermore, ROS not only induces inflammatory mediators, such as TNF-𝛼, ICAM-1, IL-6, IL-8, MCP-1, and cyclooxygenase 2 (COX-2) through NF-𝜅B activation, but also upregulates various angiogenic factors via the HIF-1 pathway, including VEGF, stromal cell-derived factor-1, angiopoietin, and erythropoietin. These changes collectively drive retinal vascular inflammation and neurodegeneration (9). Thus, oxidative stress plays a critical role in DR development and progression, and antioxidant therapies may hold promise as future treatment strategies.

Keyword co-occurrence analysis using VOSviewer classified the entire network into three clusters each representing a distinct theme. Cluster 1 (red) focused on the role of inflammation in cellular injury during DR. Clinically DR is classified into two stages: non-proliferative DR (NPDR) and PDR based on the presence of neovascularization. Severe NPDR PDR or DME are collectively defined as vision-threatening DR (44). Traditionally DR has been regarded as a microvascular disease primarily attributed to disruption of the BRB. The BRB is formed by RMECs and surrounding smooth muscle cells sealed by pericytes. In early DR RMECs and pericytes undergo apoptosis manifesting as the appearance of “ghost cells.” Subsequent proliferation of RMECs on the inner membrane contributes to BRB leakage and pathological angiogenesis (45).

Accumulating evidence now indicates that DR is not only a vascular disorder but also a neurodegenerative disease, with neuronal and glial apoptosis observed in its early stages (4). Retinal pigment epithelium (RPE) cells and white blood cells are also implicated in DR pathogenesis (46, 47). Specifically, under hyperglycemic stress, RMECs are activated to release inflammatory mediators. These inflammatory factors, in conjunction with ROS, induce a shift in microglia from an anti-inflammatory (M2) phenotype to a pro-inflammatory (M1) phenotype. Müller cells, astrocytes, and RPE cells subsequently amplify the inflammation response (6, 48). In addition, leukocytes adhere to ICAM-1 expressed on the vascular endothelium (a process known as leukostasis), thereby damaging RMECs and further compromising the BRB (47).

NF-κB has been identified as a key regulator of inflammatory protein induction in DR (40). Consequently, elevated levels of pro-inflammatory mediators—TNF-α, IL-6, IL-8, IL-1β, MCP-1, VEGF, COX-2, and iNOS sustain a chronic inflammatory state within the retina, leading to neural and vascular cell apoptosis, BRB breakdown, and angiogenesis (9, 40). Novel therapeutics targeting these inflammatory proteins have been explored in early DR, with some showing promising results (41). However, significant efforts is still necessary to translate these findings from the bench to the bedside.

### 4.3 Research frontiers

According to the keyword burst analysis, emerging research frontiers in inflammation and DR can be summarized as follows: the role of the NLRP3 inflammasome, the influence of gut microbiota, the correlation between DR and AMD, and advances in identification and optical coherence tomography (OCT).

Recent studies have highlighted that the NLRP3 inflammasome, a central component of innate immunity, is extensively activated in DR (49). Both host-derived damage-associated molecular patterns (DAMPs) generated under dysregulated glucose metabolism, such as ROS and ATP, and the chronic inflammatory milieu characteristic of DR, act as potent stimuli for NLRP3 activation (49). Once activated, the NLRP3 inflammasome promotes pyroptosis of RMECs and secretion of inflammatory mediators, including IL-6, IL-1β, and TNF-α, which induce excessive retinal inflammation and disrupt the RBR in early DR (49). Conversely, inhibition of the NLRP3 inflammasome can attenuate RMEC apoptosis and migration and downregulate AGEs-induced VEGF expression (50). Moreover, NLRP3 appears to play a crucial role in the crosstalk between RMECs and pericytes (51) and has also been implicated in DR-associated neurodegeneration, although some findings remain paradoxical (52).

Targeting the priming or activation of the NLRP3 inflammasome or the downstream signaling pathways, especially the classical NLRP3/CASP1/GSDMD axis, has yielded promising experimental results (53, 54). However, significant efforts are required to elucidate the precise role of NLRP3 in DR pathogenesis fully and to evaluate its potential as a therapeutic target in clinical practice.

The gut microbiota is a highly dynamic and complex system composed of trillions of microorganisms. Although the precise mechanisms remain unclear, the concept of the “microbiota–gut–retina axis” is increasingly accepted, suggesting that dysbiosis of gut microbiota plays a significant role in DR progression (55, 56). Both microbial imbalance, including changes in bacterial diversity, abundance, and composition, and alterations in microbial metabolites such as lipopolysaccharide (LPS), trimethylamine N-oxide (TMAO), bile acids (BAs), and short-chain fatty acids (SCFAs), may induce chronic low-grade inflammation and oxidative stress in the retina, thereby initiating or exacerbating DR (55, 56).

Distinct differences in gut microbial structure have been observed between DR patients and either healthy individuals or diabetic patients without DR (56, 57). For example, 𝛼-diversity and β-diversity are significantly lower in DR compared to controls (58). According to 16S rRNA sequencing, beneficial genera such as *Bifidobacterium* and *Lactobacillus* are found to be decreased, while pathogenic genera, including *Escherichia* and *Enterobacter,* are found to be increased in DR (59). However, potential confounding factors, including ethnicity, age, sex, diet, and medication use, should be carefully considered in future studies to ensure accurate results.

Dysregulation of the gut microbiota may disrupt the intestinal barrier, allowing bacterial components and endotoxins to enter the systemic circulation. This leakage can induce an inflammatory state by altering the levels of pro-and anti-inflammatory factors, contributing to BRB breakdown in DR (55). Additionally, microbial metabolite imbalances influence DR pathogenesis (56, 60). For instance, elevated LPS levels from Gram-negative bacteria activate the TLR–NF-κB pathway, leading to increased production of proinflammatory cytokines, such as IL-6, IL-1β, and TNF-α (55). TMAO exacerbates retinal cell dysfunction and BRB breakdown by promoting inflammation and oxidative stress (55, 61). Conversely, certain bile acids, such as tauroursodeoxycholic acid (TUDCA), have been shown to reduce retinal inflammation and vascular permeability by activating G protein-coupled receptor 5, thereby improving the pathogenesis of DR (56, 62). SCFAs can also downregulate retinal inflammation, helping to restore retinal homeostasis in DR (55).

Collectively, these findings indicate that research on the relationship between gut microbiota and DR is rapidly expanding, and modulating the gut microbiota may represent a promising strategy for the early diagnosis and treatment of DR.

AMD is another important retinal degenerative disorder and the leading cause of blindness in developed countries, constituting 8.7% of global blindness (63). AMD is classified into two forms: dry macular degeneration (dAMD), characterized by the presence of drusen between the RPEs and Bruch’s membrane and the progressive loss of RPEs and photoreceptor cells, and wet macular degeneration (wAMD), characterized by choroidal neovascularization (64). Although the pathogenesis of AMD is complex and not yet fully understood, oxidative stress and inflammation play crucial roles, as they do in DR (65). Notably, the prevalence of AMD is significantly higher in patients with DR than in those without DR (66). Both genetic and non-genetic factors, including aging, hyperglycemia, and hypertension, can disrupt retinal homeostasis and trigger inflammatory cascades, leading to vascular dysfunction and retinal cell death, and ultimately contributing to the development of AMD and DR under chronic inflammatory conditions (66).

Recent studies have highlighted the cyclic GMP–AMP synthase (cGAS)–stimulator of interferon genes (STING) pathway as a key driver of inflammation in ocular diseases, including DR and AMD (65, 67). Aberrant activation of the cGAS–STING signaling by oxidative stress, mitochondrial dysfunction, or Alu DNA has been implicated in both conditions. As a central component of innate immunity, its overactivation inevitably results in excessive inflammation and cellular degeneration (68). Currently, anti-VEGF therapy is the mainstay of treatment for severe PDR, DME, and wAMD, although its efficacy is often suboptimal (69), while effective therapies for dAMD are lacking (64, 70). Given that cGAS–STING signaling regulates multiple inflammatory pathways, inhibitors of this pathway may represent a promising treatment for DR and AMD.

Inflammation plays a crucial role in the pathogenesis of DR and AMD. Emerging therapies that target multiple factors involved in inflammation may be advanced as treatment options. Dazdotuftide, which exerts dual inhibition on TLR/NF-κB signaling and NRP-1/VEGF signaling while promoting macrophage polarization toward the anti-inflammatory M2 phenotype, has been proposed as a potential treatment for DR and both forms of AMD (71). Similarly, Palomid 529 (PBT) has demonstrated anti-inflammatory effects and therapeutic potential for treating DME and dAMD by suppressing numerous cytokines, including COX, TNF-α, and ICAMs, as well as the complement components C3 and C2 (72). Nevertheless, the development of inflammation-based therapies for DR and AMD is still far from satisfactory, and further investigation is warranted.

Researchers have recently devoted increasing attention toward the genetic susceptibility underlying the onset and progression of DR. Since inflammation plays a primary role in DR, deciphering the molecular mechanisms of inflammatory pathways holds promise for improving diagnosis and enabling personalized therapies. Variation in inflammatory genes, particularly TNF-α and IL-6, has been shown to aggravate DR (73, 74). Bioinformatics analyses further revealed that immune-related genes, such as *CCR4, CXCR6, C3AR1, LPAR1,* and *C5AR1*, are upregulated (75), while the promoters of *NLRP3*, *TGFB1, CCL2,* and *TNFSF2* are hypomethylated in DR (76). Moreover, elevated levels of pro-inflammatory proteins, including NF-𝜅B and IL-1β, can induce structural and functional changes in retina cells through protein–metabolite interactions (77).

Clinically, DR patients demonstrate an overt inflammatory phenotype, with vitreous and aqueous humor showing markedly elevated levels of cytokines and chemokines, which correlate with disease severity (11, 12). However, obtaining intraocular samples is invasive and often impractical. OCT, widely adopted in retinal imaging, provides a noninvasive method to assess retinal morphology and has recently emerged as a potential tool to visualize inflammation in DR (78). Macrophage-like cells on the human retinal surface can be visualized using OCT and adaptive optics scanning laser ophthalmoscopy (AO-SLO) (79). Under physiological conditions, microglia predominate the retinal macrophage population, with sparse perivascular macrophages and hyalocytes present within 5–10 μm of the vitreoretinal interface. During inflammation, however, monocytes and monocyte-derived macrophages become more prominent (78).

Hyperreflective foci (HRF) detected on OCT, characterized by detached, small (<30 μm diameter) moderately reflective spots mainly located in the inner and outer retina, have been recognized as a clinical biomarker of focal inflammation (80). HRF correlates with elevated levels of inflammatory cytokines in the aqueous humor (81) and poor glycemic control in DR patients (82). Recent studies have reported that HRF represents the agglomeration of activated microglial cells and may be indicative of an inflammatory state in early-stage DR, suggesting that HRF has potential as an *in vivo* biomarker for retinal neuroinflammation (80, 81, 83). Furthermore, the number of HRFs reduced rapidly following treatment with anti-VEGF drugs (81, 84), or even more with corticosteroids (85), indicating its potential for monitoring therapeutic efficacy in DR. However, the application of HRF is time-consuming and requires experienced examiners, underscoring the urgent need for automated detection methods (86, 87).

To the best of our knowledge, this work is the first bibliometric analysis of inflammation in DR. Although numerous studies have explored the association between inflammation and DR, there remains a noticeable lack of clarity regarding the overall research trends in this area. By comprehensively mapping current research hotspots and frontiers from a bibliometric perspective, our study addresses this gap. These findings not only deepen readers’ understanding of the field but also provide a valuable reference to guide international collaborations and inspire basic and clinical research in the future.

### 4.4 Limitations

This study provided an overview of the current status and emerging research frontiers on inflammation in DR. However, some limitations should be addressed. First, our analysis was restricted to publications indexed in the WOSCC database, and therefore, some relevant studies from other sources may have been overlooked. Secondly, since the WOSCC database is continuously updated, recently published high-quality literature may not have been captured due to insufficient time since publication. Third, the bibliometric and visualization analyses were performed on VOSviewer and CiteSpace, which may have introduced algorithm bias. We hope to conduct more comprehensive and in-depth research in the future.

## 5 Conclusion

Academic output in this field has markedly increased over the past decade, with accumulating evidence supporting a close relationship between DR and inflammation. The US and China have been the leading contributors to this progress. OCT-based screening and precise identification of inflammatory indicators are vital for the early diagnosis and follow-up of DR. Future research should focus on elucidating the roles of the NLRP3 inflammasome and gut microbiota in DR-associated inflammation, as well as on the development of targeted therapeutic strategies. Furthermore, investigating the shared inflammatory mechanisms underlying both DR and AMD represents an emerging and urgent frontier with significant clinical implications.
